# Integrated Network Pharmacology and Cross-Species Analysis Suggest a Potential Role of AKT1/HIF1A Axis in Shuanghuanglian for Pneumonia–Myocarditis Comorbidity

**DOI:** 10.3390/vetsci13060578

**Published:** 2026-06-12

**Authors:** Yongquan Shi, Wenwen Ding, Hongbin Duan, Hua Zhang, Panpan Sun, Kuohai Fan, Wei Yin, Jianzhong Wang, Jia Zhong, Huizhen Yang, Zhenbiao Zhang, Yaogui Sun, Hongquan Li, Na Sun

**Affiliations:** 1 Key Laboratory of Modernization of Traditional Chinese Veterinary Medicine of Shanxi Province, School of Animal Medicine, Shanxi Agricultural University, Taigu 030800, China; stone0326@hotmail.com (Y.S.); dww1030@126.com (W.D.); zhsn060511@126.com (H.Z.); sunpp0505@163.com (P.S.); dkyyinwei@126.com (W.Y.); jianzhongwang@cau.edu.cn (J.W.); zhongjia3294@163.com (J.Z.); hzyang2020@163.com (H.Y.); zbzhangvet@sxau.edu.cn (Z.Z.); dkyypb@163.com (Y.S.); livets@163.com (H.L.); 2 Animal Husbandry Service Center of Cao County, Caoxian 274400, China; 13583028636@163.com; 3 Key Laboratory of Modernization of Traditional Chinese Veterinary Medicine of Shanxi Province, Laboratory Animal Management Center, Shanxi Agricultural University, Taigu 030800, China; fkhyxj@163.com

**Keywords:** Shuanghuanglian oral liquid, heart–lung crosstalk, cross-species homology, molecular docking, ADMET, veterinary translational pharmacology

## Abstract

Shuanghuanglian oral liquid (SHL) is widely used in companion animals and poultry, but its molecular mechanism in pneumonia–myocarditis comorbidity and heart–lung inflammatory crosstalk remains largely unclear. This computational study investigated the conserved AKT1/HIF1A-mediated immunoregulatory mechanism of SHL and its cross-species translational potential in veterinary medicine. Network pharmacology was integrated with GO, KEGG, and Reactome enrichment analyses, protein–protein interaction network construction, ADMET evaluation, cross-species sequence homology analysis (human, dog, cattle, and pig), molecular docking, and molecular dynamics simulation. A total of 61 active compounds, 251 putative targets, and 52 common targets associated with pneumonia and myocarditis were identified. These targets were mainly enriched in inflammation- and immune-related pathways, including TNF, IL-17, AGE–RAGE, and PPAR signaling. AKT1 and HIF1A showed high sequence conservation across species (85–98%). Key compounds exhibited favorable binding affinity to AKT1, and molecular dynamics simulation suggested the stability of the Baicalein–AKT1 complex. ADMET analysis suggested favorable pharmacokinetic properties and low predicted toxicity. These findings suggest that SHL may potentially alleviate pneumonia and myocarditis through modulation of the conserved AKT1/HIF1A axis and support its potential as a complementary therapeutic approach for managing heart–lung inflammatory diseases in multiple livestock species. This entirely computational study highlights promising mechanisms that should be further validated in vivo.

## Simple Summary

Pneumonia and myocarditis frequently occur together in livestock and companion animals because of the close functional relationship between the lungs and heart. Shuanghuanglian oral liquid (SHL) is widely used in veterinary practice for dogs, cats, and poultry, but its role in heart–lung inflammatory interactions and its applicability to other species remain unclear. In this study, we used network pharmacology, molecular docking, and molecular dynamics simulation to explore the potential mechanism of SHL against pneumonia and myocarditis. A total of 61 active compounds and 52 common therapeutic targets were identified, with AKT1 and HIF1A emerging as important targets involved in inflammation and immune regulation. These proteins showed high sequence conservation among humans, dogs, pigs, and cattle, suggesting that SHL may exert similar effects across species. Major SHL compounds exhibited favorable binding affinity and stable interactions with the target proteins, and in silico toxicity analysis indicated favorable safety profiles. These findings suggest that SHL may have potential as a complementary therapeutic approach for the treatment of heart–lung inflammatory diseases in multiple animal species, although this entirely computational study highlights promising mechanisms that require experimental validation in vivo.

## 1. Introduction

Shuanghuanglian oral liquid (SHL), composed of Jinyinhua (Lonicerae Japonicae Flos), Huangqin (Scutellariae Radix), and Lianqiao (Forsythiae Fructus), is a traditional Chinese medicine formulation widely used in veterinary practice [1]. As a classic heat-clearing and detoxifying formulation, SHL has shown synergistic advantages in antibacterial, antiviral, anti-inflammatory, and immunomodulatory activities [2,3], with increasing application value in animal disease prevention and control, particularly for managing inflammatory diseases in livestock and companion animals [4].

The heart–lung crosstalk reflects the close physiological relationship between the respiratory and cardiovascular systems, in which pulmonary gas exchange and cardiac circulation work together to maintain systemic oxygen supply and cardiopulmonary homeostasis [5]. This interaction is increasingly recognized to involve inflammatory signaling processes. When this balance is disrupted, pulmonary inflammation may extend to systemic responses and hypoxemia that impair cardiac function, while cardiac dysfunction can further worsen lung injury and disturb gas exchange, creating a self-reinforcing pathological loop between the heart and lungs [6,7,8]. Pneumonia and myocarditis are two prevalent inflammatory diseases in veterinary clinical practice [9,10]. Inflammatory disorders of the respiratory and cardiovascular systems are closely interconnected through bidirectional heart–lung interactions. Severe pulmonary infections can trigger systemic inflammatory responses that subsequently impair cardiac function, while myocardial inflammation may further aggravate pulmonary injury through circulatory dysfunction and immune dysregulation [11,12].

**Figure 1:**
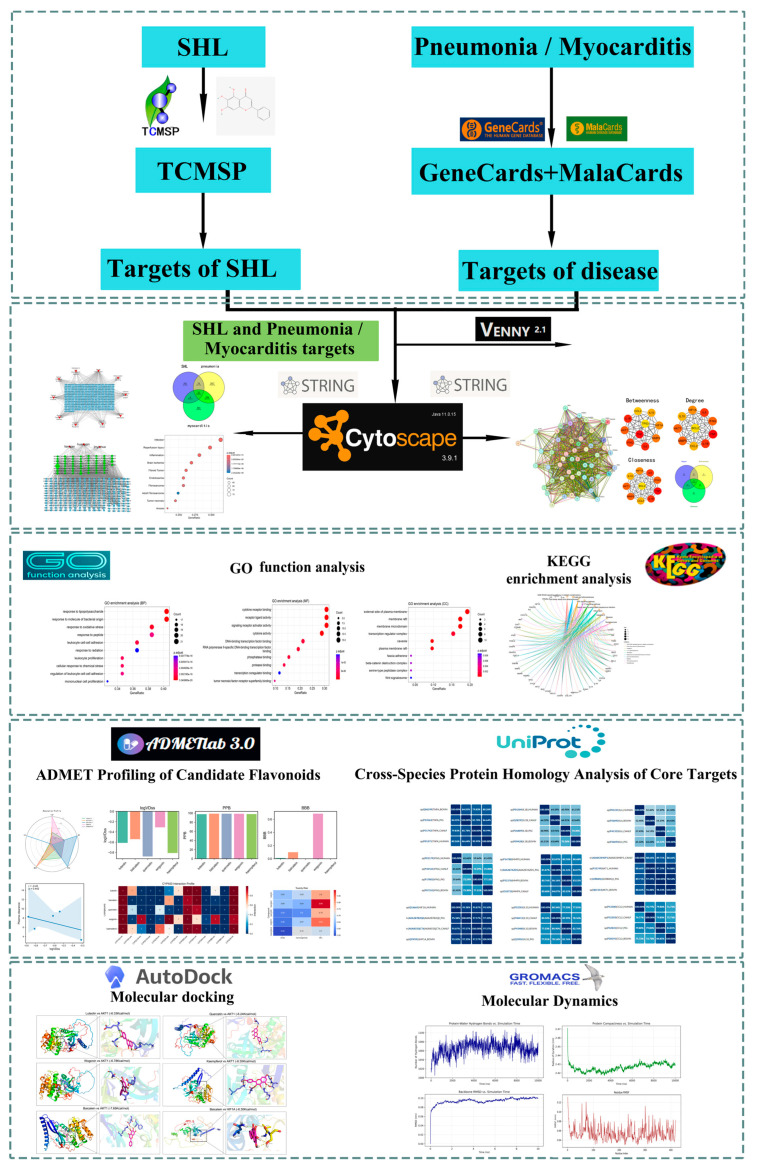
Flowchart of this study. Abbreviations: SHL: Shuanghuanglian oral liquid; TCMSP: Traditional Chinese Medicine Systems Pharmacology Database and Analysis Platform; OB: Oral Bioavailability; DL: Drug-Likeness; GO: Gene Ontology; KEGG: Kyoto Encyclopedia of Genes and Genomes; BP: Biological Process; CC: Cellular Component; MF: Molecular Function; PPI: Protein–Protein Interaction; ADMET: Absorption, Distribution, Metabolism, Excretion and Toxicity.

Network pharmacology has emerged as an effective approach for exploring the multi-component, multi-target, and multi-pathway characteristics of traditional Chinese medicine formulations, while molecular docking can further evaluate the potential interactions between bioactive compounds and target proteins at the structural level [13]. However, like other computational approaches, network pharmacology has inherent limitations, including reliance on the quality and completeness of public databases, potential bias introduced by in silico prediction algorithms, and the need for further experimental validation to confirm biological relevance [14,15]. In addition, cross-species homology analysis enables the assessment of whether pharmacological targets identified in human studies are conserved in major veterinary species, such as pigs, cattle, and dogs, providing important evidence for translating pharmacological mechanisms from human medicine to veterinary applications [16,17,18]. Based on these approaches, the present study integrated network pharmacology, molecular docking, and cross-species homology analysis to investigate the active compounds, key targets, and potential signaling pathways of SHL in the treatment of pneumonia and myocarditis [19]. This computational study aims to provide mechanistic insights into the therapeutic potential of SHL and generate hypotheses for future experimental validation, thereby offering a preliminary scientific basis for its rational application in veterinary medicine for the management of inflammatory diseases in livestock. Although SHL is included in the Chinese Veterinary Pharmacopeia with established empirical doses for dogs, cats, and chickens [20], its precise molecular mechanisms in pneumonia–myocarditis comorbidity and heart–lung inflammatory crosstalk remain insufficiently defined [21]. In addition, its potential applicability in major livestock species, such as pigs and cattle, has not been systematically evaluated. This study addresses these gaps by providing comparative veterinary pharmacological evidence to support its more rational and expanded clinical use [22]. The flowchart showing the outline of this study is presented in Figure 1.

## 2. Materials and Methods

### 2.1. Screening of SHL Active Compounds and Related Targets

SHL is composed of Jinyinhua (Lonicerae Japonicae Flos), Huangqin (Scutellariae Radix), and Lianqiao (Forsythiae Fructus). The Traditional Chinese Medicine Systems Pharmacology Database and Analysis Platform (TCMSP, https://www.tcmsp-e.com/tcmsp.php, accessed on 19 November 2025) was employed to screen compounds and drug targets for the three herbs using criteria of oral bioavailability (OB) ≥ 30% and drug-likeness (DL) ≥ 0.18. Drug target information was obtained from the UniProt database (https://www.uniprot.org, accessed on 19 November 2025) to identify corresponding drug target genes.

### 2.2. Target Genes for Pneumonia and Myocarditis

Comprehensive searches were conducted in GeneCards (https://www.genecards.org/, accessed on 19 November 2025), OMIM (https://www.omim.org, accessed on 19 November 2025), MalaCards (https://www.malacards.org/, accessed on 19 November 2025), and Therapeutic Target Database (TTD, https://ttd.idrblab.cn/, accessed on 19 November 2025) using “pneumonia” and “myocarditis” as keywords to retrieve potential target genes associated with these diseases. For the GeneCards database, disease targets with Relevance Score > 1 were selected. Access to MalaCards data was obtained under Ticket #986964. Targets collected from the four databases were merged, and duplicate entries were removed based on UniProt gene symbols to generate a non-redundant disease target set. No additional weighting or ranking strategy was applied during database integration.

### 2.3. VENN Analysis

The online tool VENNY 2.1.0 (https://bioinfogp.cnb.csic.es/tools/venny/, accessed on 19 November 2025) was utilized for intersection analysis of drug targets and disease targets to identify common targets of SHL for treating pneumonia and myocarditis. The intersection targets were used for subsequent enrichment and network analyses.

### 2.4. Enrichment Analysis

Drug targets were imported into the DAVID bioinformatics database (https://davidbioinformatics.nih.gov/, accessed on 20 November 2025) for REACTOME_PATHWAY pathway enrichment analysis, with screening criteria set at *p* < 0.05 and FDR < 0.05. Cytoscape 3.10.3 software was employed to construct drug-component-target networks for the top 10 pathways. Simultaneously, RStudio version 2024.09.0+375 was utilized to load R packages including clusterProfiler (4.18.4), org.Hs.eg.db (3.22.0), enrichplot (1.30.5), ggplot2 (4.0.2), and DOSE (4.4.0) for disease ontology (DO) analysis of drug targets, as well as Gene Ontology (GO) analysis of intersection targets between drugs and diseases, encompassing biological process (BP), molecular function (MF), and cellular component (CC) categories, along with Kyoto Encyclopedia of Genes and Genomes (KEGG) pathway enrichment analysis. Enrichment analysis screening thresholds were established at *p* value < 0.05 and Q value < 0.05. Multiple testing correction was performed using the Benjamini–Hochberg method. Bubble charts and chord diagrams were generated to visualize the top 10 ranked enrichment items.

### 2.5. Protein–Protein Interaction (PPI) Network Construction and Core Target Screening

Intersection targets between drugs and diseases were imported into the STRING database (https://cn.string-db.org, accessed on 19 November 2025) with default medium confidence score ≥ 0.4 to construct the PPI network. Although a confidence score of 0.4 is relatively moderate and may increase network connectivity, it is commonly used in network pharmacology studies to capture broader interactions while balancing sensitivity and specificity. The PPI network was exported in PNG and TSV formats, and Cytoscape 3.10.3 software was employed for core target screening. The cytoHubba plugin was used to analyze the top 10 genes based on degree centrality (Degree), betweenness centrality (Betweenness), and closeness centrality (Closeness). The top 10 genes from each centrality metric were selected, and a VENN analysis was performed to identify the overlapping core targets that ranked highly across all three metrics. These core targets were considered key regulatory nodes in the network.

### 2.6. ADMET Prediction

The pharmacokinetic and toxicity properties of five candidate compounds were evaluated using the online platform ADMETlab 3.0 (https://admetlab3.scbdd.com/, accessed 20 November 2025). Canonical SMILES structures of the candidate compounds were obtained from the PubChem (https://pubchem.ncbi.nlm.nih.gov/, accessed 20 November 2025), and submitted to the server, and key absorption, distribution, metabolism, excretion, and toxicity parameters were predicted using default settings. Evaluated indicators included molecular weight (MW), lipophilicity (logP), topological polar surface area (TPSA), MDCK permeability, volume of distribution (logVDss), plasma protein binding (PPB), blood–brain barrier permeability (BBB), interactions with major cytochrome P450 enzymes, plasma clearance, and toxicity-related endpoints including Ames mutagenicity, carcinogenicity, and drug-induced liver injury (DILI). It should be noted that all ADMET predictions were computationally derived. Predicted results were subsequently visualized and compared to assess pharmacokinetic characteristics and safety profiles of candidate compounds.

### 2.7. Cross-Species Protein Homology Comparison

Cross-species protein homology comparison was conducted to identify highly homologous proteins for guiding veterinary medication. UniProt’s Align tool was employed to compare core targets among human (Homo sapiens), dog (Canis lupus familiaris), cattle (Bos taurus), and pig (Sus scrofa). This analysis was limited to pairwise sequence identity comparisons. Although high sequence identity may indicate possible structural similarity, it cannot be directly interpreted as evidence of conserved function, expression patterns, pathway regulation, or drug response across species. Phylogenetic and structural analyses were not included in this study.

### 2.8. Molecular Docking

AutoDock Vina software version 1.1.2 was employed for molecular docking analysis of major active components of SHL with highly homologous targets for treating pneumonia and myocarditis. Target protein structures were obtained from AlphaFold 3 (https://alphafoldserver.com, accessed on 20 November 2025), and compound structures in mol2 format were retrieved from the TCMSP database. AutoDock was utilized for protein preprocessing, including removal of water molecules and addition of hydrogen atoms. Flexible docking methods were adopted for ligand–protein molecular docking, and binding energies were calculated. Lower binding energy indicates better binding, with values < −5.0 kcal/mol indicating good binding activity between ligand and target, and <−7.0 kcal/mol indicating strong docking activity. Finally, PyMOL 1.3.X software was used to visualize molecular docking results.

### 2.9. Molecular Dynamics (MD) Simulation

MD simulations were conducted using the GROMACS package to evaluate protein conformational stability. The protein structure was preprocessed in PyMOL by retaining chain A and removing crystal water molecules, heteroatoms, and redundant ligands. The simulation system was constructed using the AMBER14SB force field and TIP3P water model in a cubic box with a 1.0 nm buffer distance, followed by solvation with SPC216 water molecules and ion neutralization using Na^+^ and Cl^−^. Energy minimization was performed using the steepest descent algorithm, followed by NVT and NPT equilibration under position restraints at 300 K and 1 bar, respectively. After equilibration, an unrestrained production MD simulation of 100 ns was carried out. Trajectories were corrected by backbone fitting to remove translational and rotational motions. Structural stability and flexibility were assessed by analyzing backbone root-mean-square deviation (RMSD), root-mean-square fluctuation (RMSF), radius of gyration (Rg), and protein–water hydrogen bonds. Data visualization was performed using Python version 3.14.3 with the NumPy (2.4.1) and Matplotlib (3.10.8) packages.

### 2.10. Statistical Analysis

Statistical analysis was performed using RStudio software version 2024.09.0+375 with “limma (3.6.9)”, “DOSE (4.4.0)”, and “clusterProfiler (4.18.4)” packages. GO, KEGG, Reactome, and DO enrichment analyses were based on hypergeometric testing, with Benjamini–Hochberg correction applied to control the false discovery rate. Terms with *p* < 0.05 and FDR (Q-value) < 0.05 were considered significant. Pearson correlation was used to assess associations among ADMET parameters. In the PPI network, key nodes were identified in Cytoscape 3.10.3 based on degree, betweenness, and closeness centrality. Statistical significance was established at *p* < 0.05.

## 3. Results

### 3.1. Identification of Active Compounds and Potential Targets of SHL

**Figure 2:**
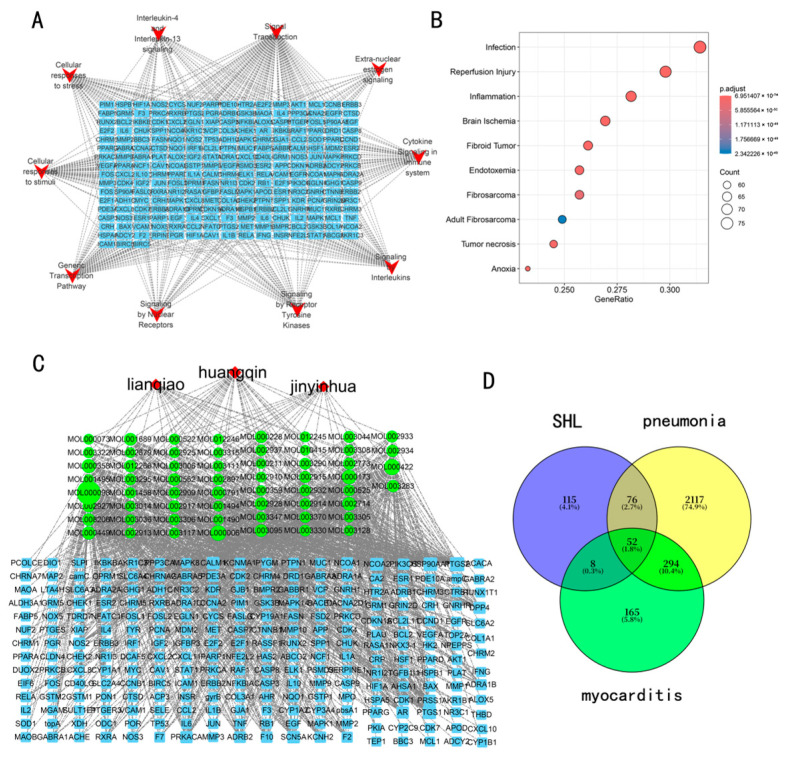
Network pharmacology analysis of SHL. (**A**) The drug–compound–target interaction network constructed from 61 active ingredients and 251 potential targets identified via TCMSP (green nodes represent compounds, blue nodes represent targets, and red nodes highlight hub compounds). (**B**) Bubble chart of DO enrichment showing the top disease categories associated with SHL targets, with bubble size proportional to gene count and color intensity indicating statistical significance. (**C**) Reactome pathway network highlighting the central immune and signal transduction modules (red nodes denote highly connected pathways). (**D**) VENN diagram illustrating the 52 overlapping targets shared among SHL, pneumonia, and myocarditis. Abbreviations: DO: Disease Ontology; PPI: Protein–Protein Interaction; SHL: Shuanghuanglian oral liquid.

The present study identified 61 active components and 251 drug targets of SHL from the TCMSP database using network pharmacology approaches (Figure 2A, Table S1). DO analysis revealed that SHL-related targets were predominantly associated with inflammation and injury responses, including reperfusion injury, infection, cerebral ischemia, inflammation, and hypoxia. Additionally, certain targets were related to tumor-associated diseases such as fibroma, fibrosarcoma, and tumor necrosis, suggesting its potential multi-target, multi-disease mechanism (Figure 2B, Table S2). The Reactome database contains relationships between signaling and metabolic molecules and their organization into biological pathways and processes. Reactome pathway enrichment analysis demonstrated that SHL-related targets were primarily enriched in immune and signal transduction-related pathways, including IL4 and IL13 signaling pathways, interleukin signaling pathways, immune system cytokine signaling pathways, receptor tyrosine kinase signaling pathways, and nuclear receptor signaling pathways. Furthermore, enriched pathways involved general transcriptional regulation, cellular responses to stimuli and stress, and extracellular estrogen signaling (Figure 2C).

### 3.2. GO and KEGG Enrichment Analysis of Overlapping Targets

**Figure 3:**
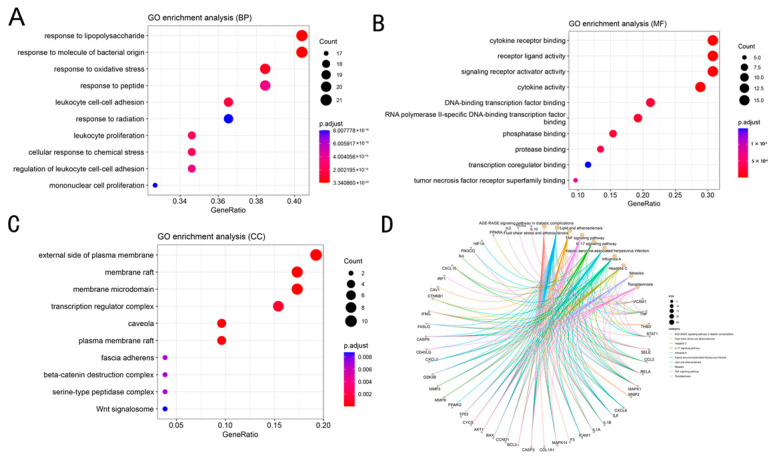
Enrichment analyses of the 52 overlapping targets between SHL and pneumonia–myocarditis. Bubble charts display the top 10 significantly enriched terms (*p* < 0.05, Q < 0.05) for (**A**) biological process (BP), (**B**) molecular function (MF), and (**C**) cellular component (CC), with bubble size representing gene count and color scale indicating adjusted *p*-value. (**D**) Chord diagram of KEGG pathway enrichment showing the relationships between core targets and the top inflammation-, immune, and metabolism-related pathways (TNF, IL-17, AGE-RAGE, PPAR, and virus infection signaling). Abbreviations: GO: Gene Ontology; BP: Biological Process; MF: Molecular Function; CC: Cellular Component; KEGG: Kyoto Encyclopedia of Genes and Genomes.

VENN analysis identified 52 intersection targets among SHL-related targets, pneumonia-associated genes, and myocarditis-associated genes (Figure 2D). Enrichment analysis results (Table S3) demonstrated that in terms of BP, core targets were primarily enriched in immune and inflammation-related processes, including response to lipopolysaccharide, response to bacterial-derived molecules, oxidative stress response, radiation response, leukocyte proliferation, monocyte proliferation, cellular response to chemical stress, and regulation of leukocyte cell adhesion (Figure 3A). These terms are closely related to excessive immune activation and inflammatory cell recruitment, which are considered important mechanisms underlying the progression from pulmonary infection to myocardial inflammatory injury. Regarding MF, targets were significantly enriched in functions including cytokine receptor binding, receptor ligand activity, receptor activator activity, and cytokine activity, additionally involving DNA-binding transcription factor binding, phosphatase binding, protease binding, and transcription regulator binding (Figure 3B), suggesting that the shared targets may participate in cytokine-mediated inflammatory communication between pulmonary and cardiac tissues. For CC, targets were predominantly localized in cell membrane-related structures, including plasma membrane outer side, membrane raft, membrane microdomain, caveolae, transcription regulator complex, and plasma membrane raft, as well as Wnt signalosome, β-catenin destruction complex, and serine-type peptidase complex structures (Figure 3C), indicating that these targets may be involved in signal transduction and inflammatory receptor activation at the cell membrane level. Pathway network analysis revealed that core targets were mainly enriched in inflammation, immune, and metabolism-related signaling pathways, including TNF signaling pathway, IL-17 signaling pathway, AGE-RAGE signaling pathway in diabetic complications, lipid and atherosclerosis-related pathways, PPAR signaling pathway, and virus infection-related pathways (Figure 3D). Although these pathways are broadly involved in inflammatory diseases, their simultaneous enrichment suggests that SHL may regulate interconnected inflammatory and oxidative stress responses shared by both pneumonia and myocarditis rather than acting through a single disease-specific pathway.

### 3.3. PPI Network Construction and Core Target Screening

**Figure 4:**
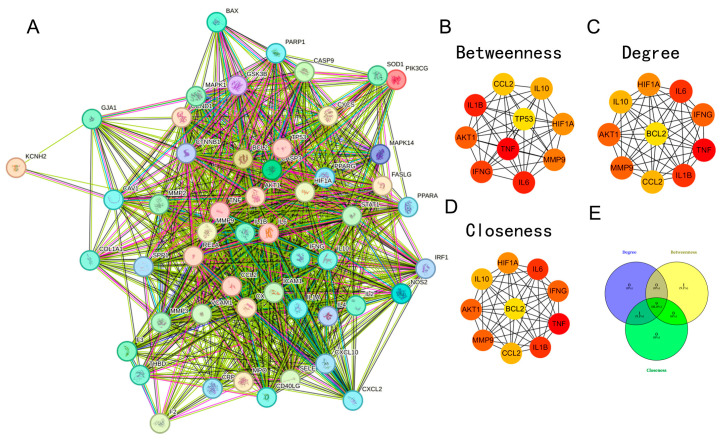
Protein–protein interaction (PPI) network and core target identification. (**A**) Full PPI network of the 52 overlapping targets generated by STRING (medium confidence ≥ 0.4), with edge thickness representing interaction strength. (**B**–**D**) Top 10 hub genes ranked by (**B**) degree centrality, (**C**) betweenness centrality, and (**D**) closeness centrality using the cytoHubba plugin in Cytoscape, and node color gradient indicates the magnitude of centrality values—darker red corresponds to higher centrality scores, while yellow represents relatively lower values within the top 10 hub genes. (**E**) VENN diagram showing the intersection of top-ranked genes across the three-centrality metrics, yielding nine core targets (TNF, IL1B, IL6, IFNG, MMP9, AKT1, HIF1A, IL10, CCL2).

PPI results demonstrated that 52 drug targets exhibited 901 edges with close interconnections between targets (Figure 4A, Table S4). The top 10 genes were screened using betweenness centrality, degree centrality, and closeness centrality (Figure 4B–D). VENN analysis identified key targets, with TNF, IL1B, IL6, IFNG, MMP9, AKT1, HIF1A, IL10, and CCL2 nodes prominent in all three centrality indicators, representing hub proteins in the network (Figure 4E), suggesting potential regulatory roles in inflammatory and immune-related signaling networks associated with pneumonia and myocarditis.

Among these, AKT1 and HIF1A were prioritized for further investigation primarily because they exhibited high network centrality and play critical roles in cell survival, hypoxic response, and inflammatory signaling pathways that are highly relevant to pneumonia–myocarditis comorbidity. Other classical inflammatory cytokines, such as TNF, IL6, and IL1B, are frequently observed in inflammation-related network analyses because of their broad roles in immune regulation and high connectivity.

### 3.4. ADMET Profiling of Candidate Flavonoids

**Figure 5:**
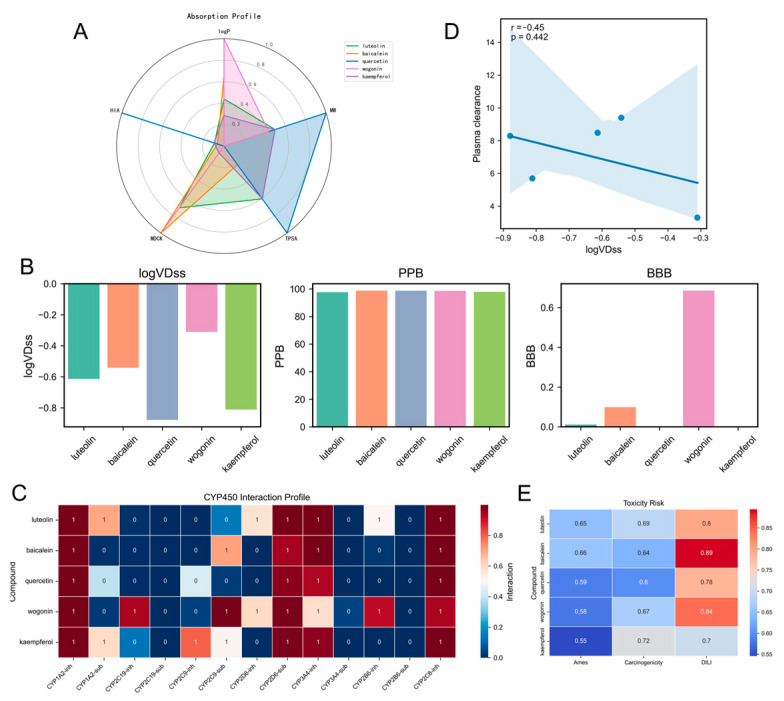
ADMET profiling of the five candidate flavonoids (luteolin, baicalein, quercetin, wogonin, and kaempferol) from SHL. (**A**) Radar plot of absorption-related physicochemical properties (logP, MW, TPSA, HIA, MDCK permeability). (**B**) Bar charts of key pharmacokinetic parameters: volume of distribution (logVDss), plasma protein binding (PPB, %), and blood–brain barrier permeability (BBB). (**C**) Heatmap of CYP450 enzyme interactions (inhibitor/substrate activity; red = high interaction probability, blue = low). (**D**) Scatter plot showing the correlation between logVDss and plasma clearance (Pearson r = −0.45, *p* = 0.442). (**E**) Toxicity risk heatmap for Ames mutagenicity, carcinogenicity, and drug-induced liver injury (DILI). Abbreviations: ADMET: Absorption, Distribution, Metabolism, Excretion and Toxicity; MW: Molecular Weight; TPSA: Topological Polar Surface Area; MDCK: Madin–Darby Canine Kidney permeability; logVDss: Logarithm of volume of distribution at steady state; PPB: Plasma Protein Binding; BBB: Blood–Brain Barrier permeability; CYP450: Cytochrome P450; DILI: Drug-Induced Liver Injury.

To further evaluate the pharmacokinetic characteristics and drug-likeness of identified flavonoids, comprehensive ADMET analysis was conducted (Table S5). Overall, the five compounds exhibited broadly comparable absorption-related physicochemical features, including molecular weight, lipophilicity (logP), topological polar surface area (TPSA), and predicted MDCK permeability (Figure 5A). Distribution analysis indicated moderate predicted tissue distribution across compounds, while plasma protein binding was consistently high (>95%), suggesting strong binding affinity in circulation (Figure 5B). Among candidates, wogonin demonstrated relatively higher predicted blood–brain barrier permeability compared to other flavonoids. CYP450 interaction profiling revealed potential interactions with several major metabolic enzymes, particularly CYP1A2 and CYP3A4, indicating these pathways may contribute to their metabolic processing (Figure 5C). Predicted excretion parameters varied among the analyzed compounds, reflecting potential differences in pharmacokinetic behavior (Figure 5D). Toxicity prediction suggested an overall favorable safety profile for compounds, although baicalein exhibited relatively higher predicted risk for drug-induced liver injury (DILI) compared to other candidates (Figure 5E).

### 3.5. Cross-Species Protein Homology Analysis of Core Targets

**Figure 6:**
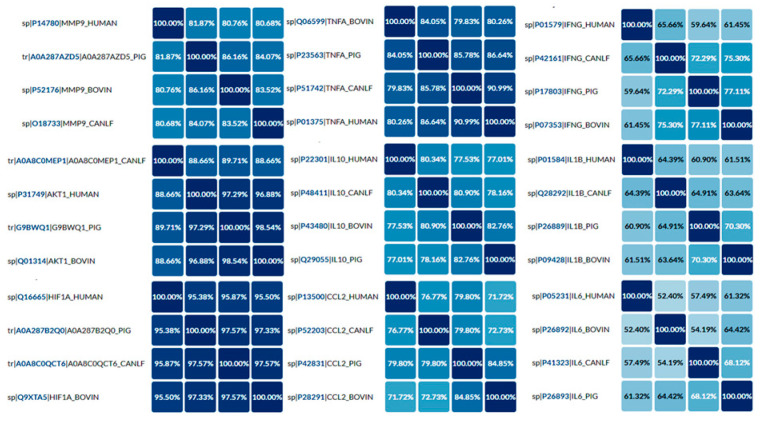
Cross-species sequence identity heatmap of the core target proteins. Pairwise amino acid sequence homology (%) is shown among human (Homo sapiens), dog (Canis lupus familiaris), cattle (Bos taurus), and pig (Sus scrofa). AKT1 and HIF1A exhibit the highest conservation (85–98%), while IL6 shows the lowest (50–68%). Color intensity scales from light blue (lower homology) to dark blue (higher homology). Abbreviations: Homo sapiens: Human; Canis lupus familiaris: Dog; Bos taurus: Cattle; Sus scrofa: Pig.

Sequence homology analysis of core targets was compared across human (Homo sapiens), dog (Canis lupus familiaris), cattle (Bos taurus), and pig (Sus scrofa) species (Table S6). The results showed variable degrees of conservation among different targets, with AKT1 and HIF1A exhibiting highest cross-species homology (85–98%), IL6 showing lowest homology (50–68%), and remaining targets displaying moderate similarity (60–85%) (Figure 6). The high sequence conservation of AKT1 and HIF1A among humans and major livestock species suggests potential similarities in their structural features and biological roles across species. Combined with their prominent positions in the PPI network, these two targets were selected for subsequent molecular docking analysis. However, sequence homology alone does not necessarily indicate conserved regulatory pathways or identical pharmacological responses, and experimental validation is still required.

### 3.6. Molecular Docking of Active Compounds to Key Targets

The protein structures used for molecular docking were obtained from AlphaFold3 and evaluated before docking analysis. AKT1 showed a high overall confidence score (average pLDDT = 83.06), with high-confidence predictions in the kinase domain and ATP-binding pocket regions (Supplementary Figure S1A). HIF1A had a moderate overall confidence score (average pLDDT = 63.66), while the core bHLH-PAS functional domains still maintained relatively high confidence. Lower confidence scores were mainly distributed in the flexible C-terminal regions, which is consistent with the structural features of transcription factors (Supplementary Figure S1B). Overall, the core functional regions of both proteins are considered sufficiently reliable for docking simulations.

**Figure 7:**
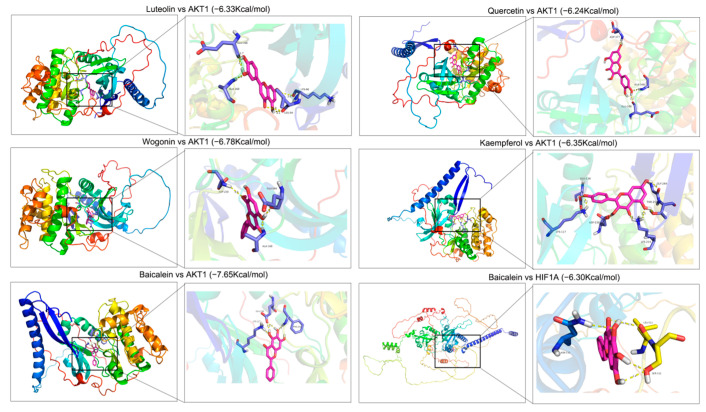
Molecular docking visualization of major active compounds from SHL with the core targets AKT1 and HIF1A. Representative binding poses are shown for luteolin, quercetin, wogonin, kaempferol, and baicalein.

Molecular docking results indicated that all compounds displayed favorable binding affinities toward AKT1, with binding energies below −6.0 kcal/mol. Among these, baicalein displayed the lowest binding energy with AKT1 (−7.65 kcal/mol), indicating relatively stronger predicted binding compared with the other ligands rather than absolute highest affinity. In comparison, baicalein’s binding energy with HIF1A was −6.30 kcal/mol, weaker predicted interaction than with AKT1. Meanwhile, luteolin, quercetin, wogonin, and kaempferol could stably bind with AKT1 (Figure 7).

### 3.7. Stability Analysis of the Baicalein–AKT1 and HIF1A Complex Based on MD Simulation

**Figure 8:**
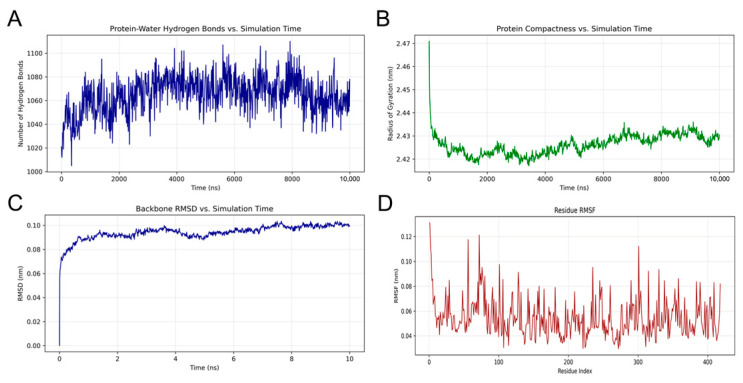
MD simulation analysis of the baicalein–AKT1 complex. (**A**) Protein–water hydrogen bonds during the simulation. (**B**) Radius of gyration (Rg) of AKT1. (**C**) Backbone root-mean-square deviation (RMSD) of the Baicalein–AKT1 complex. (**D**) Root-mean-square fluctuation (RMSF) of AKT1 residues. The results indicate that the Baicalein–AKT1 complex remained structurally stable throughout the simulation. Abbreviations: MD: Molecular Dynamics; Rg: Radius of Gyration; RMSD: Root-Mean-Square Deviation; RMSF: Root-Mean-Square Fluctuation.

The MD simulation results demonstrated that the Baicalein–AKT1 and HIF1A complexs maintained excellent overall conformational stability throughout the simulation process. The number of protein–water hydrogen bonds remained relatively stable with moderate fluctuations, indicating dynamic interactions with the surrounding solvent environment (Figure 8A). The radius of gyration (Rg) rapidly decreased during the initial equilibration stage and subsequently stabilized within a narrow range around 2.42–2.43 nm, suggesting that the overall compactness of AKT1 remained stable upon ligand binding (Figure 8B). The backbone RMSD gradually increased during the early stage of the simulation and eventually stabilized at approximately 0.09–0.10 nm, indicating that the complex reached a stable conformational equilibrium without significant structural deviation (Figure 8C). RMSF analysis showed that most residues exhibited relatively low fluctuation amplitudes, while only several loop and terminal regions displayed relatively higher flexibility, suggesting that the core structure of AKT1 remained stable during the simulation (Figure 8D). MD simulation of the HIF1A protein showed overall conformational stability during the 100 ns trajectory (Supplementary Figure S2), with no major structural deviations observed in RMSD, Rg, or RMSF profiles. Overall, these results suggest that the system reached a stable equilibrium state during the simulation, reflecting the structural stability of the protein–ligand complex under physiological-like conditions.

## 4. Discussion

This study represents the first comparative veterinary pharmacology investigation of SHL targeting the heart–lung inflammatory crosstalk in pneumonia and myocarditis. Through an integrated approach combining network pharmacology, molecular docking, ADMET profiling, and cross-species protein homology analysis, we suggest that SHL may exert potential regulatory effects through pathways involving the AKT1/HIF1A signaling axis. The identification of 52 overlapping targets, enrichment in TNF, IL-17, AGE-RAGE, and PPAR pathways, and favorable docking affinity of baicalein toward AKT1 provide computational evidence supporting the potential multi-component and multi-target characteristics of SHL.

The finding that AKT1 and HIF1A exhibit 85–98% sequence homology across humans, dogs, cattle, and pigs is of particular veterinary significance. AKT1, a central regulator of cell survival and anti-apoptotic signaling [23,24], and HIF1A, a key transcription factor in hypoxic adaptation, have both been implicated in inflammatory and cardiopulmonary diseases [25,26]. From a translational veterinary pharmacology perspective, the high degree of cross-species conservation observed for AKT1 and HIF1A may suggest possible conservation of related signaling mechanisms across species rather than supporting direct extrapolation of pharmacological effects from human studies to veterinary species. Conserved signaling pathways may therefore represent potential candidates for future cross-species pharmacological investigation, particularly in livestock where species-specific pharmacological data remain limited [16,27,28]. Previous studies have shown that overactivation of HIF-1A may promote iNOS expression and excessive nitric oxide production, which impairs mitochondrial respiration and contributes to cytopathic hypoxia [29]. Pneumonia and myocarditis are frequently associated with local hypoxia and metabolic imbalance during their pathological progression. Activation of the AKT1 and HIF1A may enhance cellular adaptation to hypoxic stress, reduce oxidative injury, and modulate immune responses, thereby potentially improving disease progression [30]. In the present study, enrichment and docking analyses suggested possible involvement of AKT1- and HIF1A-related pathways without providing evidence of functional activation, inhibition, or direct target engagement. In contrast, members of the interleukin family showed relatively lower cross-species homology (50–70%). This variability may be related to host–pathogen coevolution, as immune-related genes are often subject to strong selective pressures that drive rapid evolution and functional diversification [31,32]. Our findings indicate that SHL may help disrupt this pathological cycle through the regulation of key inflammatory mediators, while also influencing metabolic homeostasis and immune responses via PPAR signaling [33]. These modulatory effects support the concept of a heart–lung axis in acute respiratory syndromes and porcine respiratory disease complexes, in line with recent veterinary observations.

The addition of ADMET profiling further provides preliminary pharmacokinetic insights that may support the evaluation of the translational potential of our findings. The five candidate flavonoids (baicalin, luteolin, quercetin, wogonin, and kaempferol) exhibited predicted absorption and distribution profiles, high plasma protein binding, and acceptable CYP450 interactions, with an overall low predicted toxicity risk. Although baicalin showed a slightly elevated DILI prediction, its relatively favorable docking results and reported anti-inflammatory activity still position it as representative candidate compound rather than a confirmed leading pharmacodynamic component [34,35,36]. These pharmacokinetic data partially address a common limitation of network pharmacology studies and provide preliminary reference information for future veterinary formulation development. The compounds analyzed in this study were screened from the TCMSP database rather than identified directly from SHL preparations. Therefore, their actual concentrations and in vivo exposure in veterinary formulations remain uncertain. Further studies incorporating chemical profiling and pharmacokinetic evaluation are needed.

Compared with previous SHL research mainly focused on human respiratory infections or animal models [4,37], our study extends current work by incorporating cross-species conservation analysis and exploring potential the lung–heart inflammatory interactions in major livestock species. The high homology of AKT1/HIF1A may suggest conserved biological relevance across species, but does not directly support the “from human to animal” translational strategy. Therefore, the cross-species applicability of SHL is not fully established at the molecular level. Accordingly, SHL may represent a potential adjunct therapeutic strategy, and its translational relevance requires cautious interpretation in the context of antimicrobial resistance and drug residue concerns in intensive farming systems.

Although SHL is already listed in the Chinese Veterinary Pharmacopeia with recommended doses for companion animals and poultry, its molecular mechanism in the heart–lung inflammatory crosstalk and its potential application in major economic livestock have not been systematically investigated. The present study represents an initial effort to provide hypothesis-generating evidence through an integrated computational approach, suggesting the AKT1/HIF1A axis as potential key target, in line with previous reports on AKT1-centered flavonoid networks in acute lung injury and network pharmacology-based analyses of polyphenols in pulmonary diseases [38,39]. These findings may help explain the clinical efficacy observed in practice and provide a preliminary molecular basis for expanding SHL use in swine and bovine respiratory-cardiac diseases, offering a safe and sustainable alternative to antibiotics amid growing concerns of resistance and drug residues. This study is entirely computational. Although the integration of ADMET and cross-species homology enhances reliability, experimental validation is essential to confirm the predicted efficacy and safety of SHL.

### Limitations

This study was based entirely on computational analyses and lacks direct experimental evidence. The predicted targets and pathways were mainly derived from TCMSP and other public databases, which may introduce incomplete coverage and potential bias. The active compounds were not experimentally confirmed in the SHL formulation used in this study, and their concentrations and pharmacokinetic characteristics in target animal species were not evaluated. Although AlphaFold3 models with relatively high confidence scores were used for docking, predicted structures cannot fully substitute for experimentally resolved proteins. In addition, high sequence similarity across species does not necessarily reflect conserved biological regulation or equivalent pharmacological responses. Several hub genes identified in this study are also common regulators in inflammatory networks and may not be specific to pneumonia–myocarditis interactions. The ADMET results were likewise prediction-based and were not validated in target animal species. Taken together, these results provide preliminary computational insights into the potential mechanisms of SHL, while additional experimental studies are still needed to determine their biological and translational relevance.

## 5. Conclusions

In conclusion, this entirely computational study suggests that SHL may potentially attenuates pneumonia and myocarditis through modulation of the conserved AKT1/HIF1A axis within the lung–heart inflammatory network. The favorable docking scores, acceptable ADMET properties, and high cross-species homology collectively indicate promising multi-target mechanisms worthy of further investigation. These findings are hypothesis-generating and provide a preliminary basis for further research. Future in vitro and vivo trials and clinical studies in target animal species are strongly recommended to translate these computational insights into practical veterinary medicine.

## Data Availability

These data were derived from the following resources available in the public domain：TCMSP: https://tcmspw.com/tcmsp.php; GeneCards: https://www.genecards.org; OMIM: https://www.omim.org; MalaCards: https://www.malacards.org; Therapeutic Target Database (TTD): https://ttd.idrblab.cn/.

## Supplementary Materials

The following supporting information can be downloaded at https://www.mdpi.com/article/10.3390/vetsci13060578/s1, Table S1: Molecule-Target Gene Association Table. Table S2: Disease Enrichment Analysis Results of Target Genes of SHL. Table S3: Functional Enrichment Analysis Results of Target Genes. Table S4: PPI Network Dataset of Target Genes. Table S5: Druggability and Molecular Feature Dataset of Active Molecules from SHL. Table S6: Multi-Species Protein Sequence Dataset of Core Target Genes. Figure S1: AlphaFold 3-predicted structures and confidence assessment of AKT1 and HIF1A. Figure S2: Molecular dynamics simulation of the HIF1A protein.

## Abbreviations

The following abbreviations are used in this manuscript:

ADMET	Absorption, Distribution, Metabolism, Excretion, and Toxicity
BBB	Blood–Brain Barrier
BP	Biological Process
CC	Cellular Component
DILI	Drug-Induced Liver Injury
DL	Drug-Likeness
DO	Disease Ontology
FDR	False Discovery Rate
GO	Gene Ontology
KEGG	Kyoto Encyclopedia of Genes and Genomes
MD	Molecular Dynamics
MDCK	Madin–Darby Canine Kidney
MF	Molecular Function
MW	Molecular Weight
OB	Oral Bioavailability
PPI	Protein–Protein Interaction
PPB	Plasma Protein Binding
SHL	Shuanghuanglian Oral Liquid
TCMSP	Traditional Chinese Medicine Systems Pharmacology Database and Analysis Platform
TPSA	Topological Polar Surface Area
