# Supplementary material for: Integrated Network Pharmacology and Cross-Species Analysis Suggest a Potential Role of AKT1/HIF1A Axis in Shuanghuanglian for Pneumonia–Myocarditis Comorbidity
Source: Vet Sci. 2026 Jun 12;13(6):578. doi: 10.3390/vetsci13060578 (PMC13307895; doi:10.3390/vetsci13060578)
Supplement: Supplementary file 1 [file vetsci-13-00578-s001.zip › Supplementary Figures.pdf]

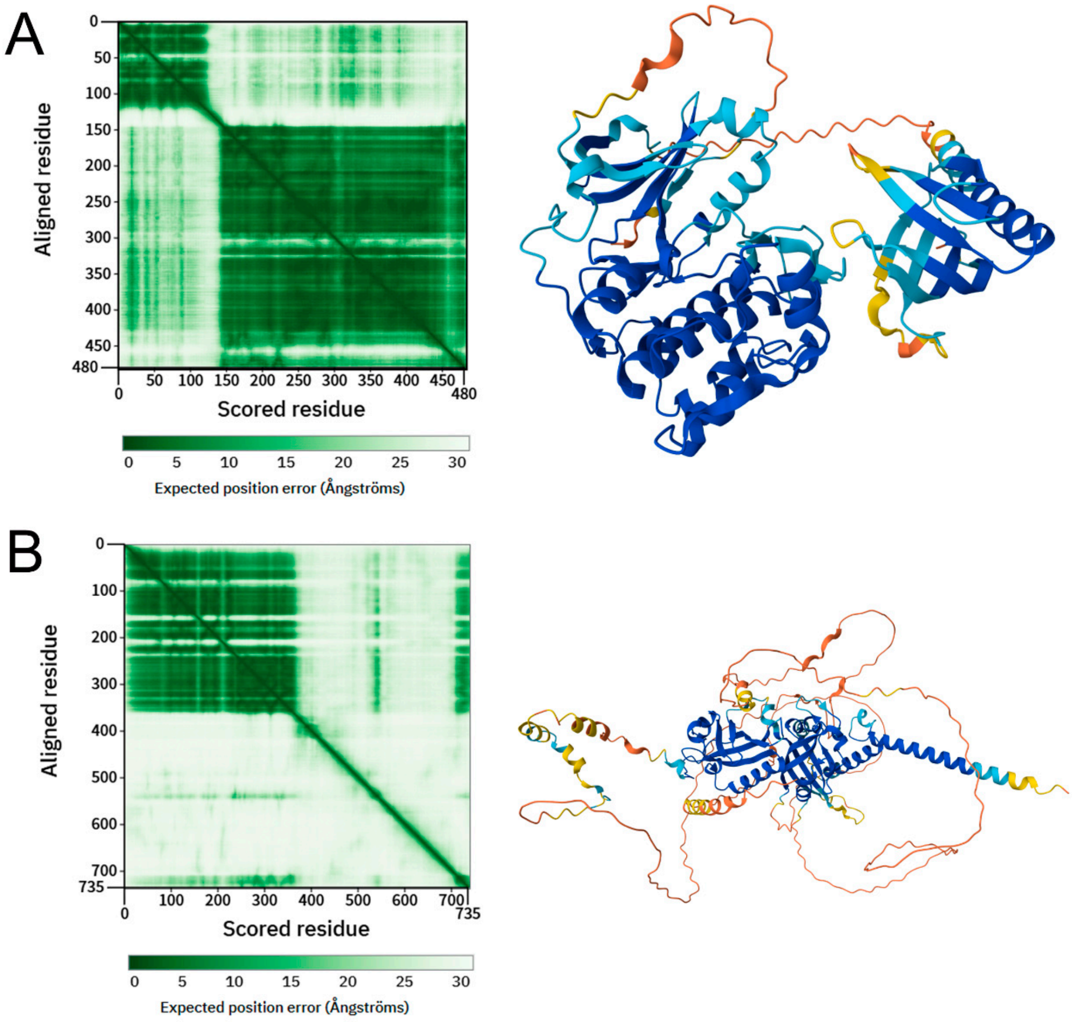

Supplementary Figure S1. AlphaFold 3-predicted structures and confidence assessment of AKT1 and HIF1A. (A) AKT1. Left: pLDDT confidence map and three-dimensional structure colored according to pLDDT scores. (B) HIF1A. pLDDT confidence map and three-dimensional structure colored according to pLDDT scores. Blue: high confidence; red: low confidence. Abbreviations: pLDDT: predicted Local Distance Difference Test.

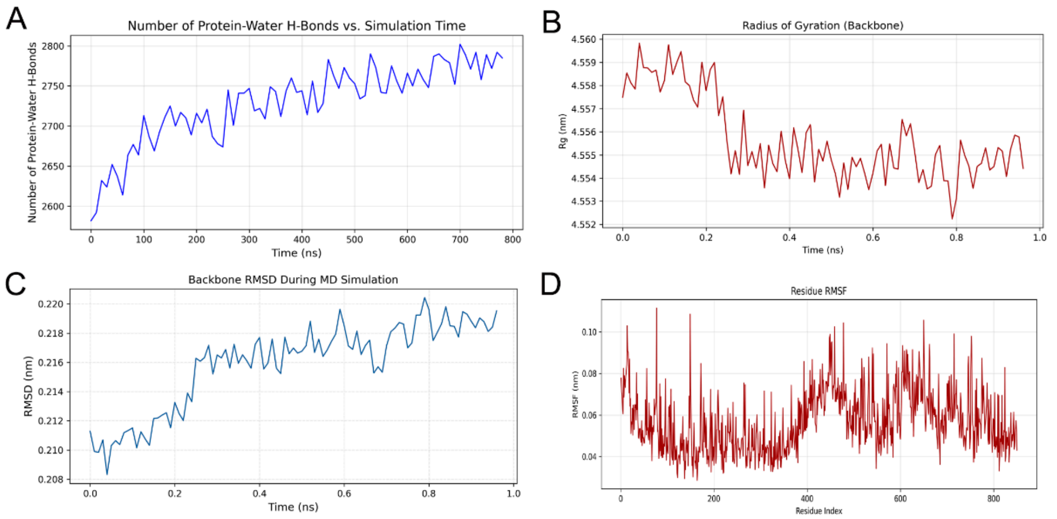

Supplementary Figure S2. Molecular dynamics simulation of the HIF1A protein. (A) Protein–water hydrogen bonds during the simulation. (B) Radius of gyration (Rg) of HIA1A. (C) Backbone root-mean-square deviation (RMSD) of the complex. (D) Root-mean-square fluctuation (RMSF) of HIF1A residues. Abbreviations: MD: molecular dynamics; RMSD: root-mean-square deviation; RMSF: root-mean-square fluctuation; Rg: radius of gyration.
